# SUNTEL pilot study: first school-based integration of the ugly duckling sign and a dedicated e-learning platform with peer education for melanoma prevention

**DOI:** 10.3389/fonc.2025.1665136

**Published:** 2025-10-09

**Authors:** Ignazio Stanganelli, Serena Magi, Elisabetta Costa, Matelda Medri, Franca Gentilini, Chiara Scutellà, Daniela De Zerbi, Giorgia Ravaglia, Silvia Gerosa, Federica Zamagni, Chiara Doccioli, Sara Gandini, Maria Costanza Cipullo, Saverio Caini

**Affiliations:** ^1^ Skin Cancer Unit, IRCCS IRST Romagnolo Institute for the Study of Tumors “Dino Amadori”, Meldola, Italy; ^2^ School of Dermatology, Department of Medicine and Surgery, University of Parma, Parma, Italy; ^3^ Graphic Design and Multimedia “Good Sun Good Skin” project, Istituto Oncologico Romagnolo, Forlì, Italy; ^4^ IOR Scuola, Istituto Oncologico Romagnolo, Forlì, Italy; ^5^ Scientific High School “Alfredo Oriani”, Ravenna, Italy; ^6^ Emilia-Romagna Cancer Registry, Romagna Unit, IRCCS IRST Romagnolo Institute for the Study of Tumors “Dino Amadori”, Meldola, Italy; ^7^ Clinical Epidemiology Unit, Institute for Cancer Research, Prevention and Clinical Network (ISPRO), Florence, Italy; ^8^ Department of Experimental Oncology, IEO European Institute of Oncology IRCCS, Milan, Italy; ^9^ Directorate General for Student Affairs, Inclusion, and Career Guidance, Prevention and Health Education Unit, Ministry of Education, Rome, Italy; ^10^ Cancer Risk Factors and Lifestyle Epidemiology Unit, Institute for Cancer Research, Prevention, and Clinical Network (ISPRO), Florence, Italy

**Keywords:** melanoma, e-prevention, peer education, secondary school, ugly duckling

## Abstract

**Introduction:**

Educational initiatives among young people are pivotal in promoting healthy behaviors, yet only a few studies to date have used peer education in schools to convey health messages for melanoma prevention. Here we report the results of a peer-education-based skin cancer prevention program combined with teledidactics (SUN Education and TELematic Learning – SUNTEL) targeting high school students in Italy.

**Methods:**

In 2023-2024, thirty third-year high school students attended training sessions to become peer educators and then conducted interventions across first-year students using a multimedia platform. Students targeted by the peer-education intervention completed pre- and post-intervention questionnaires to assess changes in their knowledge, attitudes and behaviours regarding melanoma prevention. Marginal homogeneity tests were used to compare responses in the pre- vs. post-intervention questionnaires.

**Results:**

The study population included 323 students (56.7% boys) mostly aged 14–16 years. Most participants had fair/light dark skin (>97%); 24% had freckles, and over one third developed skin rash often/always after UV exposure. Students’ knowledge about melanoma risk factors and prevention, and attitude about UV exposure, changed significantly after the intervention, e.g., students wanting to use a tanning bed/lamp dropped from 9.6% to 3.8% (p-value <0.001), and those aware that using a tanning bed/lamp is as risky as tanning in the sun rose from 75.4% to 90.5% (p-value <0.001). Knowledge of the ABCDE rule rose from 7.5% to 96.6%; for the ugly duckling sign, the change was from 9.1% to 91.2%.

**Conclusions:**

e-learning/web-based tools and peer-education proved highly effective in enhancing students’ knowledge regarding melanoma prevention and recognition, proving comparably or even more effective compared to teachers-/physicians-led educational approaches. Notably, this is the first school-based program to introduce the “ugly duckling” sign, a simple and reproducible rule for early melanoma detection. Sustained message reinforcement and parental involvement will be key to achieving lasting behavioral change in sun safety.

## 1 Introduction

Melanoma is a highly aggressive skin cancer with an increasing global incidence (1). Early detection and preventive measures are crucial in reducing both morbidity and mortality associated with this malignancy. Educational initiatives, particularly among young people, play a pivotal role in raising awareness and promoting behaviors that mitigate risk factors. The use of digital tools, and particularly, their increasing integration into the educational system, has shown potential for the effective dissemination of health education activities. Skin cancer prevention is among the key points emphasized by the European Code Against Cancer (ECAC) (2), with a specific focus on the school population. Scientific evidence suggests that education programs conducted in high school can be effective in favourably modifying the students’ knowledge, beliefs and attitudes on skin cancer prevention, particularly when they incorporate evidence-based teaching strategies for maximum impact (3).

Peer Education is one of the most widely used approaches for engaging young people in prevention, giving them an active role. It originated as an educational methodology in the USA in the 1960s, and has since then been widely adopted in the Anglo-Saxon world for several public health prurposes (e.g. HIV prevention). Peer education programs are becoming inceasingly popular in school settings, possibly as a result of teenagers preferring to turn to their friends for assistance with health-related issues rather than adults or experts. Numerous health domains are covered by peer education proposals, emphasizing how this concept provides a versatile tool that can be adapted to different educational contexts by improving student engagement and understanding. The most recent review of school-based peer education interventions, published in 2022 by Dodd and colleagues and updated until October 2020 (4), included 73 publications from countries around the world but failed to find evidence of peer education programs in the field of skin cancer prevention. Most included studies assessed treatments that addressed drinking, smoking, or substance use (n = 16), promotion of healthy lifestyles (n = 17), or sex education/HIV prevention (n = 23). Numerous programs show indications of efficacy, suggesting that peer education could be a viable approach to school health improvement. Of note, evidence was stronger with regard to the potential of peer education programmes to bring about favourable changes in health-related knowledge, while somewhat weaker for changes in health-related behavior.

In the setting of skin cancer prevention, the most recent literature review covered 25 studies published during 1946–2020 and encompassing a total of 22,683 adolescent participants. Of these, 16 studies reported a significant increase in knowledge, 21 studies demonstrated favourable changes in behavior, and 15 studies showed significant changes in attitudes (5). Despite limitations due to the heterogeneity of the studies, these findings underline the importance of multidimensional active learning strategies as essential components of melanoma prevention and early diagnosis. Among the studies included in this review, however, only one (by Loescher et al., 2019 (6)) used peer education as the primary method to convey health messages, despite its recognition as a fundamental element of health education, especially in high schools. More recently, Hanna and colleagues tested the “SunSafe Student Ambassador Program” in two Australian secondary schools and showed a statistically significant improvement in attitudes, knowledge and behaviours towards sun safety using peer-to-peer learning targeting adolescents (7). The peer-led, train-the-trainer model proved effective and the authors suggested it could be effectively adapted to a wider student age range.

In this article, we present the results of a peer-education-based skin cancer prevention program targeting adolescents, innovative in that it attempts to help fill the current knowledge gap in this research field. In detail, through the use of a multifaceted online platform and multimedia approach, we demonstrated a pilot project for an innovative e-learning program that allowed involved students both to gain training and to serve as peer educators for their schoolmates. The program called SUNTEL (SUN Education and TELematic Learning) consists of sequential steps, illustrated in greater deatil in what follows, including *(a)* an online course; *(b)* training between peer educators; *(c)* lessons on primary and secondary skin cancer prevention provided by peer educators to their high school classmates; and *(d)* evaluation of the program outcomes. SUNTEL was developed as an evolution of the multimedia program *“Il Sole per Amico for Young”*, integrating the experience gained from oncology-focused teledermatology prevention with a structured peer education methodology. Notably, this is the first known school-based program to introduce the “ugly duckling” sign, as a core educational component for adolescents, aiming to promote both prevention and early self-recognition of suspicious lesions.

## 2 Materials and methods

### 2.1 The online course “Il Sole per Amico for YOUNG”

This multimedia prevention programme was developed as part of the wider range of projects developed within the Italian Melanoma Intergroup (IMI) and targeting primary and secondary schools. It serves as an integral component of the national health education contest ‘IL SOLE PER AMICO – LET’S LEARN TO PROTECT OUR SKIN,’ carried out in collaboration with the Italian Ministry of Education. The “*Il Sole per Amico for YOUNG*” e-learning program (in English: “The sun as a friend for YOUNG”) was developed to primarily target the educational setting, particularly both pupils/students and teachers of secondary school. It focuses on health education, particularly regarding skin cancer prevention, and is delivered via the program online platform (https://soleperamico.melanomaimi.it/); a schematic structure of the web platform is provided in the **Supplementary Table 1**. This prevention programme is founded on several key components:

- robust scientific rationale;
- formal agreement with the Italian Ministry of Education (detailed in a specific protocol);
- use of existing multimedia scientific content designed for teachers, students and families at school level;
- use of educational material created in previous years by students in several schools that had participated in the “Il Sole per Amico” school contest;
- iconographic, textual, and virtual morphing content from theMelanoma Multimedia Education (MelaMEd) programme (https://www.imi-melamed.it/) and and health education school activities led by the Istituto Oncologico Romagnolo (IOR), a no profit association located in Emilia Romagna Region (North-Eastern Italy) (8).

### 2.2 Peer education: rationale and implementation training

Peer Education involves assigning trained peers the role of “influencers” within their peer group. Sharing similar attitudes, language, and experiences, these individuals are seen as credible messengers, even when discussing health-related topics. In this model, students are at the center of the educational process: “peer educators” guide and support their fellow students, or “peer learners,” using simple, direct, and effective language to explore crucial aspects of health education together. The core of the Peer Education experience is therefore the sharing of knolwegde and experiences. Once trained, educators convey what they have learned to their peers in their own unique way, creating a positive cycle of knowledge-sharing and health message dissemination. This proactive method is further enhanced through interactions with adults, allowing deeper understanding and refinement of health concepts (4).

In the 2023–2024 school year, the Peer Education project was implemented as part of the dermatology curriculum at the “Liceo Scientifico Oriani” (a secondary high school specializing in scientific subjects) in Ravenna, north-east Italy. Thirty third-year students in a biomedical biology course participated, integrating the “*Il Sole per Amico for YOUNG*” multimedia platform. In April 2024, 18 students (mostly aged 16 years) volunteered to attend structured training sessions to become peer educators. Nine students were excluded due to their non-participation in the peer education program, as they opted not to take on additional commitments due to academic workload.

The training of peer educators followed a structured multi-session program. In the first meeting, the students selected as peer educators were introduced to the principles of peer education and the role of the peer educator, with a focus on fostering self-awareness, individual strengths, and coping strategies for potential challenges. Group activities were designed to promote engagement, trust, and collaboration. Subsequent sessions focused on scientific content related to sun protection and melanoma prevention. The students were guided through the “Il Sole per Amico for Young” e-learning platform and tasked with designing their own peer-led sessions. Working in small groups, they prepared customized interventions using interactive tools and creative communication strategies integrated to “*Il Sole per Amico for YOUNG*” multimedia platform, with the aim of delivering tailored messages to younger students. A simulation session allowed peer educators to rehearse their delivery, reflect on possible classroom dynamics, and manage anxiety or self-doubt. Each group presented their planned activities and anticipated potential barriers, such as classroom resistance or performance anxiety. The final phase of the training involved logistical planning and pairing of peer educators. Over two school days in early May 2024, they carried out their educational sessions across fifteen first and second year classes. A post-session feedback form was completed by participants.

### 2.3 Parental consent, school authorization, and ethic approval

Participation was voluntary, requiring parental consent. Prior to the program, each participating school’s Institutional Review Board issued a formal communication to families, detailing the project and including a non-adherence form for those opting out. The SUNTEL programme was endorsed by the Italian Melanoma Intergroup and was approved by the ethical committee of the Romagnolo Institute for the Study of Tumors (IRST) “Dino Amadori”, Meldola, Italy.

### 2.4 Students survey

Students targeted by the peer-education programms (i.e. “peer learners”) completed pre- and post-training questionnaires (the latter, three weeks after the completion of the sessions) to assess the impact of the program on their behaviours and attitudes for melanoma prevention. The questionnaire is based on previous studies that have analyzed behaviors related to sun protection and the use of tanning bed (8), and consists of several sections each targeting specific topics relevant for skin cancer risk and prevention. In general, the questionnaire was designed to gather insights into the students’ knowledge and habits with reference with cancer risk and prevention, with particular reference to sun exposure (by determining the students’ awareness of the dangers of natural and artificial UV radiation, including the risk associated with sunburns) and early recognition of suspicious lesions (by measure the students’ understanding of self-assessment tools like the ABCDE rule and the “Ugly duckling” concept). Of note, re-administering the the questionnaire at the end of the peer education program served the purpose of assessing the effectiveness of the educational campaigns in improving sun safety awareness, thus ultimately aiding in devising future sun protection strategies.

Pre- and post-intervention paper-based questionnaires were distributed systematically during school hours by peer educators and one of the co-authors (DD). Peer learners completed the surveys at their desks, requiring approximately 10–15 minutes. All forms were collected immediately after completion to minimize data loss. The questionnaire’s clarity and comprehensibility were pre-tested by the School’s Institutional Review Board through structured feedback sessions with a small student group. This process ensured that peer learners could reliably interpret each question.

A summary of the content of each questionnaire section is as follows:

- Phenotypic Factors (5 questions): this section focuses on individual characteristics affecting melanoma risk, such as skin type, nevi presence, and sun exposure reactions;
- UV Exposure Habits (12 questions): section gathering data on exposure to natural sunlight and tanning beds, to identify risky behaviors and monitor changes after educational interventions;
- Parental Influence (3 questions): it examines parents’ role in promoting sun protection, adherence to guidelines, and knowledge transmission to their children;
- Knowledge of ABCDE and Ugly Duckling Rules (4 questions): this section tests the students’ awareness of early melanoma signs using self-assessment tools like the ABCDE rule and the “ugly duckling” concept to identify suspicious lesions. The ABCDE rule is a dermatological tool used to evaluate moles and skin lesions for potential signs of melanoma. It is an acronym for Asymmetry, Border irregularity, Color variation, Diameter greater than 6 mm, and Evolving characteristics (such as changes in size, shape, or color over time) (9). The ABCDE rule has proved useful not only for clinicians but also for educating the general population, as it provides an easy method to assess skin lesions. To enhance its relevance for early melanoma detection, we added specific items on the “ugly duckling” sign, who demonstrated good interobserver agreement on this visual recognition strategy (8). The “ugly duckling” sign highlights skin lesions that appear noticeably different from an individual’s other moles, prompting early dermatological consultation, crucial for melanoma detection. Combined with the ABCDE rule, it strengthens public education efforts, empowering individuals to conduct self-examinations and identify suspicious lesions at an early stage (10).
- Socioeconomic Characteristics (7 questions): this section explores how family socioeconomic status impacts access to sun protection resources and sun exposure habits.

### 2.5 Statistical analysis

Descriptive analyses were conducted by reporting percentage responses. Distributions were compared between the pre- and post-intervention surveys by applying the marginal homogeneity test. Considering that only a limited proportion of students failed to complete both questionnaire, a complete case analysis was conducted with no imputation of missing data.

## 3 Results

A total of 332 students were involved. Nine students were not included in the analyses because they filled in only the pre-intervention (n=7), only the post-intervention (n=1), or neither (n=1) questionnaire. The study population consists therefore of 323 students (56.7% boys and 43.3% girls), whose main characteristics are listed in **Table 1**. The majority of students had Italian citizenship (96.9%), and the age at pre-intervention questionnaire was ≤14, 15 or ≥16 years in 37.0%, 46.6%, and 16.5% of the study population, respectively. The highest education attained by the student’s parents was university degree or above (57.8% of mothers and 45.7% of fathers) and higher secondary school (37.6% of mothers and 44.1% of fathers). The large majority of the students’ parents had a paid occupation, office work being the most commonly reported employment type (37.4% of mothers and 28.6% of fathers) (**Table 1**).

**Table 1:** Distribution (N, %) of selected characteristics of participating students (n=323): demographics, citizenship, and parents’ school level and occupation.

Students’ characteristics	N	%
Sex		
male	183	56.7%
female	140	43.3%
Age		
≤14 years	119	37.0%
15 years	150	46.6%
≥16 years	53	16.5%
Citizenship		
Italian	312	96.9%
other	10	3.1%
Mother’s school level		
lower secondary or below	13	4.0%
higher secondary	121	37.6%
university degree or above	186	57.8%
other	2	0.6%
Father’s school level		
lower secondary or below	30	9.3%
higher secondary	142	44.1%
university degree or above	147	45.7%
other	3	0.9%
Mother’s occupation		
office worker	120	37.4%
teacher	58	17.7%
manual work, labourer	8	2.5%
entrepreneur/businesswoman/executive	63	19.7%
other paid occupation	46	14.3%
no paid occupation	27	8.4%
Father’s occupation		
office worker	92	28.6%
teacher	10	3.1%
manual work, labourer	50	15.5%
entrepreneur/businessman/executive	108	33.5%
other paid occupation	58	18.0%
no paid occupation	4	1.3%

In terms of phenotypic characteristics, most students had fair (61.9%) or light dark (26.0%) skin; dark blonde/light brown (44.9%) or dark brown/black (47.4%) hair; and light brown (37.8%) or dark brown/black (35.3%) eye colour (**Table 2**). Around one fourth of students had freckles (13.6% “a few”, 9.3% “some”, and 2.2% “many”), and over a third reported developing skin rash “often” (29.7%) or “always” (6.8%) after exposing to sunlight.

**Table 2:** Distribution (N, %) of phenotypic characteristics and sun sensitivity of study participants.

Students’ phenotype and sun sensitivity	N	%
Skin colour		
very fair	29	9.0%
fair	200	61.9%
light dark	84	26.0%
dark	10	3.1%
Hair colour		
red	4	1.2%
light blonde	21	6.5%
dark blonde, light brown	145	44.9%
dark brown, black	153	47.4%
Eye colour		
light blue, gray, light green	33	10.2%
dark blue, dark green	54	16.7%
light brown	122	37.8%
dark brown, black	114	35.3%
Freckles		
many	7	2.2%
some	30	9.3%
a few	44	13.6%
none	242	74.9%
Sunburn after sun exposure		
never	44	13.6%
seldom, sometimes	161	49.8%
often	96	29.7%
always	22	6.8%

The proportion of students having ever participated in the health education lab rose from 2.8% to 68.8% between the pre- and post-survey as detailed in **Table 3**, which also reports about changes in the students’ beliefs, attitudes and behaviours regarding tanning and melanoma prevention. In general, we observed no changes in reported behaviours (e.g. time spent in the sun, use of sunscreens, and use of tanning beds/lamps), which is not surprising given that the pre- and post- surveys were only about three weeks apart. Instead, there were significant changes in the students’ knowledge about melanoma prevention and attitude about exposure to UV radiation. Namely, the proportion of students that declared they wanted to use a tanning bed/lamp dropped from 9.6% to 3.8% (p-value <0.001). In addition, a larger proportion of students became aware that using a tanning bed/lamp does not reduce the risk of sunburn (84.2% to 93.6%, p-value <0.001) and is as risky as tanning directly in the sun (75.4% to 90.5%, p-value <0.001). In the post-intervention survey, the students thinking that it is worth getting badly burned to get a tan was 6.9% (significantly dropping from 12.9%, p-value <0.001).

**Table 3:** Students’ beliefs and attitudes (N, %) towards tanning and melanoma prevention in the pre- and post- surveys.

Students' beliefs and attitudes	Pre-intervention survey		Post-intervention survey		*p-value*
	N	%	N	%	
Have you ever been to the melanoma and/or prevention health education lab at your school?					
yes, I have	9	2.8%	220	68.8%	
no, I haven’t	311	97.2%	100	31.3%	***<0.001***
On average, how long do you spend in the sun without interruption when you do outdoor activities?					
less than 1 hour	21	6.6%	21	6.6%	
1–3 hours	187	58.8%	189	59.4%	
3–5 hours	82	25.8%	82	25.8%	
more than 5 hours	28	8.8%	26	8.2%	0.731
Do you usually use sunscreen when doing outdoor activities?					
always	22	6.9%	24	7.5%	
most of the time	55	17.2%	51	16.0%	
sometimes	81	25.4%	93	29.2%	
never or almost never	82	25.7%	80	25.1%	
only in summer	79	24.8%	71	22.3%	0.116
Have you used tanning lamps/beds in the last two years?					
yes	1	0.3%	1	0.3%	
no	319	99.7%	319	99.7%	NA
Would you like to use a tanning lamp/bed?					
yes	30	9.6%	12	3.8%	
no	282	90.4%	300	96.2%	***<0.001***
Have your parents used tanning lamps/beds in the last two years?					
yes	18	5.8%	19	6.1%	
no	293	94.2%	292	93.9%	0.564
Have your parents used tanning lamps/beds in the past?					
yes	28	9.5%	27	9.2%	
no	267	90.5%	268	90.8%	0.317
Do you think using a tanning bed/lamp before sun exposure helps reduce the risk of sunburn?					
yes	49	15.8%	20	6.4%	
no	262	84.2%	291	93.6%	***<0.001***
Do you think that tanning using a lamp is less risky than tanning directly in the sun?					
yes	78	24.6%	30	9.5%	
no	239	75.4%	287	90.5%	***<0.001***
Do you wear sunglasses at the beach or when doing outdoor activities?					
yes	112	35.1%	107	33.5%	
no	207	64.9%	212	66.5%	0.096
Do you like being very tanned?					
yes	209	65.5%	209	65.5%	
no	110	34.5%	110	34.5%	0.998
Do you think it’s worth getting badly burned to get a tan?					
yes	41	12.9%	22	6.9%	
no	277	87.1%	296	93.1%	***<0.001***
Do you know the ABCDE rule of melanoma?					
yes	24	7.5%	309	96.6%	
no	296	92.5%	11	3.4%	***<0.001***
What does the ABCDE rule of melanoma mean? A suspicious mole is:					
asymmetric, irregular border, heterogeneous color, diameter >6 mm, rapid evolution	123	38.4%	306	95.6%	
aseptic, banal, uniform color, diameter 3 mm, evasive	16	5.0%	3	0.9%	
symmetrical, regular border, uniform color, diameter <6 mm, stable over time	4	1.3%	7	2.2%	
I don’t know	177	55.3%	4	1.3%	***<0.001***
Do you know the “ugly duckling” sign?					
yes	29	9.1%	290	91.2%	
no	289	90.9%	28	8.8%	***<0.001***
What does the “ugly duckling” sign mean in identifying a suspicious mole?					
a mole similar to the others in shape and color	2	0.5%	2	0.6%	
a mole that “stands out” from other moles due to its shape and color	209	49.9%	292	91.5%	
a mole that hurts	3	0.7%	1	0.3%	
I don’t know	205	48.9%	24	7.5%	***<0.001***

Statistical significant p-values (at the 0.05 threshold) are highlighted in bold.

Before the program, only 7.5% of students were familiar with the ABCDE rule; this percentage rose to 96.6% in the post-peer education assessment (**Table 3**). The percentage of correct answers on the meaning of the ABCDE rule at the pre-training questionnaire was 38.4%, which increased to 95.6% after the program. Likewise, only 9.1% of students reported being aware of the “ugly duckling” sign before the educational program, which rose to 91.2% thereafter. Prior to training, 49.9% of students correctly identified the definition of the “ugly duckling” sign, whereas after training, 91.5% of students were able to accurately recognize its definition.

In terms of overall satisfaction, 94% of the adolescents who participated in the sessions led by peer educators reported that the classroom atmosphere was quite or very pleasant, and 92% found it quite or very enjoyable. The participants especially appreciated the absence of adults during follow-up activities, which allowed them a space to freely express their opinions. When asked about whether the sessions were productive, 73% of participants reported having gained new knowledge on the subject, while 27% already possessed extensive knowledge on the topics that were covered. The peer educators also expressed satisfaction with the program, finding it valuable not only for the students they educated but also as a rewarding personal growth experience.

## 4 Discussion

Melanoma is a highly aggressive form of skin cancer. Over the past 50 years, the incidence of cutaneous melanoma has progressively increased in most Caucasian populations worldwide. Approximately 85% of new skin melanoma cases each year occur in the populations of North America, Europe, and Oceania (11). In Italy, melanoma is currently the second (among men) and third (among women) most common malignancy in the population below 50 years of age (12). Early detection enables timely intervention, reducing morbidity and mortality. Moreover, the adoption of preventive behaviors - such as avoidance of excessive sun exposure, sun protection, and regular self-examinations - can significantly reduce the risk of developing melanoma and other skin cancers. School-based interventions have a positive impact on knowledge and sun protection behaviors.

The effects were greater for multicomponent interventions and young people represent a critical target group for these efforts (3), because adolescents and young adults are more likely to engage in outdoor activities and tanning behaviors, making them particularly vulnerable to UV exposure, a primary risk factor for melanoma. By targeting young audiences with early and accurate information about melanoma risks and the importance of skin health, we can influence long-term behaviors that support both prevention and early detection - not only among students themselves but also within their families. At the same time, parental modeling and communication regarding sun protection may significantly influence adolescents’ attitudes and habits. Therefore, we have emphasized the value of developing shared educational tools that directly engage families in prevention strategies. This approach is supported by literature demonstrating that parental behavior and attitudes toward sun exposure are critical determinants of youth practices, including sunscreen use, shade-seeking behavior, and avoidance of indoor tanning (3). We strengthened the presentation of how SUNTEL integrates both primary and secondary prevention. Unlike most peer education programs that focus on promoting general healthy behaviors, SUNTEL uniquely targets sun safety and melanoma prevention. It encourages protective habits (e.g., sunscreen use, avoiding tanning beds) while also promoting early detection skills through the ABCDE rule and the “ugly duckling” sign. This dual approach empowers students to adopt sun-safe behaviors and recognize suspicious lesions, filling a gap noted in previous reviews such as Dodd et al. (2022), which did not report peer-based models addressing skin cancer prevention or early self-assessment. Since then, only a few studies have adopted peer education despite its recognized value in adolescent health promotion. Among them, Loescher et al. (2019) implemented the “Students are Sun Safe” (SASS) program in underserved rural Arizona schools using a participatory research framework and peer educators trained through online modules, leading to significant and sustained improvements in sun protection knowledge and behaviors (6). Similarly, Hanna et al. evaluated the “SunSafe Student Ambassador Program” in Australia, where trained student ambassadors educated their peers, increasing awareness of melanoma risk factors and improving sunscreen use (7). The Australian model is characterized by training high school students through collaborative, in-person workshops who then educate their peers within the same school environment. The Arizona model, by contrast, involves university students as peer educators who receive formalized training with integrated digital media and teach younger students in schools. This reflects differences in educational settings and resources, with Australia emphasizing peer-to-peer learning within secondary schools, and Arizona leveraging university-level expertise to reach school-age populations. These studies highlight the value of peer education and set the stage for “SUNTEL”, among the few peer education-based melanoma prevention projects conducted to date to our knowledge. Moreover the Italian SUNTEL project emphasizes the role of third-year students as “influencers” within their peer group, leveraging their credibility and recent experience to communicate effectively with first-year students. The approach combines digital learning with direct, age-proximate peer interaction integrated with the multidimensional web platform *Il Sole per Amico for YOUNG* and aims at bridging a critical gap in adolescent skin health education.

Furthermore the positive outcomes observed in the SUNTEL project are consistent with findings from other innovative cancer education initiatives. For instance, Budzik et al. (13) demonstrated a significant increase in cancer-related knowledge among high school students following structured, teacher-led sessions in the OncoAcademy project. While OncoAcademy employed traditional didactic methods, SUNTEL introduced a novel model that combines peer education with digital learning, addressing both primary and secondary prevention goals. In the broader context of digital health, the value of multimedia and personalized education in cancer prevention is increasingly evident. Westerlinck et al. (14) developed CANRISK, a mobile health application that integrates a validated cancer risk model to inform the public about both modifiable and non-modifiable risk factors. The app provides personalized risk assessments, educational content, and behavioral guidance demonstrating the potential of technology-driven tools to actively engage individuals in their own preventive care. Together, these projects highlight a shared priority: the need to institutionalize cancer prevention education, particularly during adolescence, by leveraging both peer-driven strategies and digital platforms. Well-designed interventions—whether implemented in schools or through mHealth tools—can significantly enhance awareness, shift perceptions, and foster long-term protective behaviors. **Table 4** summarizes the salient characteristics of selected studies from the literature review that included a pre-post intervention comparison of educational programs, to facilitate comparison with our own findings.

**Table 4:** Changes in students’ beliefs and attitudes (%) obtained by educational programs with pre- and post-training questionnaires: comparison with the SUNTEL pilot study.

First Author (year)	Students (No)	Educational support	ABCDE knowledge	Tanning as prevention	Tanning as attractive	Sunbed use	Sunglasses use	Sunscreen use
Geller et al. (2005)	344	school teachers	27.1% ➢ 59.7%	–	–	–	–	–
Wu et al. (2019)	1573	researchers	–	–	–	2% ➢ 2%	23% ➢ 27%	10% ➢ 16%
Sumen et al. (2015)	567	researchers	–	–	33.9% ➢ 24.7%	–	66.6% ➢ 77.6%	41.4% ➢ 23.9%
Kouzes et al. (2017)	100	school teachers	–	73.6% ➢ 64.3%	57.5% ➢ 60.2%	5.8% ➢ 8.2%	46% ➢ 55.1%	49.4% ➢ 60.3%
Cassel et al. (2018)	208	school teachers	–	76% ➢ 72%	76% ➢ 70%	–	4.1% ➢ 1.9%	–
Loescher et al. (2019)	220	high school peers	18.8% ➢ 81.3%	–	–	6.2% ➢ 0%	37.5% ➢ 50%	68.8% ➢ 75%
Hanna et al. (2022) ^(a)^	218	high school peers	2.8%➢30.7%	–	25.6% ➢ 27%	2.8%➢2.8%	–	82.4%➢83.9%
	235		3.4% ➢9.5%		26.6% ➢ 23.2%	8.5%➢6.8%		84.2%➢80.9%
Stanganelli et al. (this study)	323	high school peers	38.4% ➢ 95.6%	15.8% ➢ 6.4%	65.5% ➢ 65.5%	0.3% ➢ 0.3%	35.1% ➢ 33.5%	49.5% ➢ 52.7%

a Two schools were involved in the study by Hanna et al. (2022).

### 4.1 Primary prevention

Primary melanoma prevention is based on adopting proper sun exposure behaviors, such as using SPF sunscreen, wearing sunglasses, and avoiding exposure during the peak hours of the day. In general, we observed no significant changes in reported behaviors in our study, likely due to the fact that the pre- and post-surveys were conducted only about three weeks apart. Regarding attitudes and behaviour in our population, our study found that 65.5% of students enjoyed being very tan. This attitudinal data did not change after the educational program. In Kouzes et al. (15), a similar trend is observed, with 57.5% of students considering themselves to look better with a tan before the training, increasing to 60.2% after the intervention. Likewise, in Cassel et al. (16), there is an increase in the proportion of students who view themselves as more attractive with a tan (24% pre-training and 30% post-training). Conversely, Sumen et al. (17) show a decrease in the percentage of students preferring tanned skin (from 33.9% to 24.7%). Despite the high percentage of students in our studied who declared enjoying having a deep tan, protective behaviors are more widely adopted after the training period. This might suggest that the aesthetic preference for tanning does not necessarily translate into indiscriminate and dangerous sun exposure behaviors. In fact, peer training increased, alebit slightly, the percentage of students who apply sunscreen during outdoor activities (from 49.5% to 52.7%).

Data regarding the use of sunglasses are practically stable (35.1% to 33.5%). We also reported an increase in the percentage of students aware that using a tanning bed before sun exposure does not reduce the risk of sunburn, from 84.2% to 93.6%. In two other non-peer education studies, this percentage varied from 24% to 28% (16) and from 26.4% to 35.7% (15). Moreover, our peer education intervention proved effective in improving knowledge about the importance of avoiding burns: the percentage of students who believed it was worth getting badly burned to achieve a tan decreased from 12.9% before the training to 6.9% after the training.

### 4.2 Artificial UV radiation

Our education project proved effective in improving students’ knowledge regarding the use of tanning beds, as the proportion of students who declared they wanted to use a tanning bed dwindled by nearly two-thirds after the intervention (from 9.6% to 3.8%). It is noteworthy that our study revealed that only one student (0.3%) reported using artificial lamps in the past two years, a result that remained unchanged in the follow-up questionnaire. Similarly, in the study by Wu et al., the percentage remained stable, maintaining a higher absolute value of 2% both before and after the intervention (18). Conversely, in the study by Kouzes, the percentage of users actually increased (5.8% before and 8.2% after) (15). In Australia, despite strict regulations in force, students continue to use tanning beds, with only a minimal impact (<2% of students) observed after peer education in the two schools of the SSAP project (7). Whereas in the other study employing the peer education the effectiveness of this educational approach was confirmed, with a significant reduction in tanning bed use (6.2% before and 0% after) (6). It is evident that the percentage of tanning bed use in our population is lower than that reported in the aforementioned studies. Furthermore, this figure appears even more reduced when compared to a study conducted on a similar population in Italy in 2016. In fact, in this study, 7% of students reported the use of tanning beds despite the enforcement of Legislative Decree No. 62 of May 19, 2011, which prohibits minors from using sunbeds (8). Regarding the use of tanning lamps by parents, our study revealed that slightly more than 9% of the parents of the students involved had used tanning lamps in the past, and only 6% having done so in the last two years. This figure appears lower compared to a study conducted on a similar Italian population in 2013, where the percentage of tanning lamp users was 20% (19), and it is even lower compared to the 44.7% reported in a 2015 study conducted in Italy on parents of primary school students (20).

### 4.3 Secondary prevention - ABCDE and ugly ducking

In relation to the knowledge component, we specifically considered prevention aspects by analyzing familiarity with the ABCDE rule and the “ugly duckling” sign. Before the program, only a minority of students were familiar with the ABCDE rule, while in the post-peer education assessment, the nearly majority of them (96.6%) had gained knowledge on the topic. The percentage of correct answers on the pre-training questionnaire was 38.4%, which increased to 95.6% after the program. In reference to the study by Geller et al., where educational support was provided by school teachers, the percentage of correct responses was 27.1% before and 59.7% after (21). In previous studies using peer education in high schools, the percentage of correct responses on the ABCDE rule increased from 18.8% to 81.3% in the SASS program (6) and from 2.8% to 30.7% and 3.4% to 9.5% in the two schools of the SSAP program in Australia (7). Italian high school students demonstrated the highest baseline knowledge of the ABCDE rule and showed significant improvement after e-learning education, achieving outstanding results (**Table 4**). As far as we know, our project was the first to inroduce the concept of the “ugly duckling” in school-based education programs on an international basis. Before the educational program, only 9.1% of students reported being aware of the “ugly duckling” sign; this percentage rose to 91.2% following the peer education activity. Prior to training, 49.9% of students correctly identified the definition of the “ugly duckling” sign, whereas after training, 91.5% of students were able to accurately recognize its definition. This criterion had never before been applied in school programs, where secondary prevention was limited to the ABCDE rule alone. The “ugly duckling” sign is particularly useful for identifying atypical moles that deviate from a patient’s typical nevus pattern. Increased awareness of this principle likely contributed to improved recognition of melanoma. When combined with the ABCDE rule, the ugly duckling sign may become a powerful tool for public health campaigns too. Therefore, the introduction of the “ugly duckling sign” in the SUNTEL program represents an innovative and more reproducible signature in the teaching of secondary prevention in schools, making self-examination of moles more accessible and straightforward for young people. These remarkable outcomes in both the ABCDE rule and the Ugly Duckling sign may be attributed to the innovative multimedia and multidimensional approach used in the training program. In addition to the strengths that we have mentioned, our work also has some limitations that is important to acknowledge. Specifically the three-week interval between pre- and post-tests limits the ability to assess long-term behavioral changes. Some degree of social desirability bias may be present (as usual in this type of investigations), considering that responses were self-reported in a peer-led environment. A longer follow-up period would be needed for more robust conclusions; an updated data collection among study participants is being planned at the time of manuscript writing, and will be the subject of a follow-up manuscript. Finally, the questionnaire we used is similar to others used in the literature, but has never been formally validated.

Despite increased knowledge, aesthetic preferences for tanning remained largely unchanged, with around two-thirds of students still enjoying being very tan after the program. This highlights a gap between knowledge acquisition and behavioral or attitudinal shifts. The studies referenced in the systematic review exhibit significant methodological variability, limiting comparability. This study was conducted in a single high school with a demographically homogeneous student population, which may limit the external validity and broader applicability of the findings. Further multicenter investigations are warranted to confirm the generalizability across diverse educational and socio-cultural settings. Moreover further limitations may be related to limited analysis of peer education’s impact, lack of qualitative data (e.g., student perceptions or barriers to behavior change) and finally the connection between parental behaviors and student outcomes is not explored in depth. The lack of a control group limits causal inference and future studies will include comparison groups led by teachers or dermatologists. However relevance on melanoma prevention among adolescents addresses a critical public health issue, the evidence-based approach supported by up-to-date and relevant reference and the the integration of data from multiple studies such as a comparative analysis with other studies strengthens the findings by offering context and benchmarks and provides a comprehensive overview of the field. Moreover, the use of peer education is a noteworthy and underutilized strategy that has shown promising results in skin cancer prevention. This study contributes meaningfully to the growing literature by integrating digital tools, adolescent engagement, and a structured educational methodology tailored to school environments.

### 4.4 Conclusion

Uniquely, this is the first known school-based program to incorporate the “ugly duckling” sign as a core educational tool, promoting both primary prevention and early self-recognition of suspicious lesions. Peer education proved highly effective in enhancing students’ knowledge regarding melanoma prevention and recognition, proving comparably or even more effective compared to other school-based educational approaches involving teachers or dermatologists. Furthermore, the model’s combination of digital training platforms and novel prevention tools may enhance primary prevention and early detection capabilities, while empowering students with practical skills for skin health monitoring. This structure uniquely integrates digital learning with in-person peer education within the high school environment, setting it apart from other school-based educational approaches.These encouraging results must be tempered with the awareness that repeated messaging are probably necessary to shift deeply rooted beliefs, such as the perceived attractiveness of tanning and habits related to sun exposure. Sustained reinforcement of these health messages, paired with the involvement of parents, may be essential to drive long-lasting changes in sun safety behaviors and perceptions among high school students.

## Data Availability

The raw data supporting the conclusions of this article will be made available by the authors, without undue reservation.
