# Supplementary material for: SUNTEL pilot study: first school-based integration of the ugly duckling sign and a dedicated e-learning platform with peer education for melanoma prevention
Source: Front Oncol. 2025 Oct 9;15:1665136. doi: 10.3389/fonc.2025.1665136 (PMC12545009; doi:10.3389/fonc.2025.1665136)
Supplement: Supplementary file 1 [file Table1.docx]

**Supplementary Table 1**. Structure and content of the “*Il Sole per Amico for YOUNG*” web platform (in English: “The sun as a friend for YOUNG”) (<https://soleperamico.melanomaimi.it/>).

| E- LEARNING EDUCATIONAL PROGRAM  “**IL SOLE PER AMICO**” WEB PLATFORM | ***Contents***:  1. Basic knowledge of ultraviolet radiation  2. Basic understanding of the skin as an organ  3. Interaction between skin and UVR  4. The beneficial effects of the sun  5. The harmful effects of the sun  6. Nevi and melanomas  7. Primary prevention  8. Secondary prevention  9. Dermatological assessment  ***Chapters***:  1. Epidemiology  2. Risk Factors  3. How to Recognize a Suspicious Nevus?  4. Nevi and Melanomas: What Are They?  5. The 10 Most Frequently Asked Questions  - “DIGITAL GAME” (Nevi vs Melanoma).  -“PEER EDUCATION” area  -“SKIN LAB” area |
| --- | --- |
